# Morphological diversity of the tricuspid posterior leaflet affects surgical complexity for control of tricuspid regurgitation

**DOI:** 10.1186/s13019-022-01762-5

**Published:** 2022-02-16

**Authors:** Takumi Kawase, Yosuke Takahashi, Kenta Nishiya, Noriaki Kishimoto, Kokoro Yamane, Yoshito Sakon, Akimasa Morisaki, Hiromichi Fujii, Toshihiko Shibata

**Affiliations:** Department of Cardiovascular Surgery, Osaka City University Graduate School of Medicine, 1-4-3 Asahimachi, Abeno, Osaka 545-8585 Japan

**Keywords:** Residual tricuspid regurgitation, Tricuspid valve, Two posterior leaflets

## Abstract

**Objective:**

We investigated the effect of morphological diversity of the tricuspid valve with multiple posterior leaflets on the technical outcomes of tricuspid valve repair.

**Methods:**

From April 2016 to November 2020, 141 patients were diagnosed with secondary tricuspid regurgitation associated with left heart disease and underwent tricuspid valve repair. We retrospectively analyzed the clinical and echocardiographic data of patients who underwent both preoperative and postoperative transthoracic echocardiography. We divided the patients into two groups according to the surgical technique used to treat tricuspid regurgitation: ring annuloplasty alone (Group 1, n = 109) or additional approximation of leaflet edges (edge-to-edge repair) with ring annuloplasty (Group 2, n = 32). We measured the morphological diversity of the tricuspid valve during the operation in all patients.

**Results:**

The preoperative tricuspid regurgitation score was higher in Group 2 than in Group 1 (2.1 ± 0.78 vs. 1.6 ± 0.7, respectively; *p* = 0.0046), and Group 2 contained more patients with two posterior leaflets than Group 1 [20 (63%) vs. 36 (33%), respectively; *p* = 0.003]. The univariate and multivariate logistic regression analyses showed that the presence of two posterior leaflets was an independent risk factor for additional procedures during tricuspid valve repair (odds ratio, 2.6; 95% confidence interval, 1.1–6.1; *p* = 0.033).

**Conclusions:**

Additional procedures to reduce tricuspid regurgitation were required more frequently in patients with two posterior leaflets of the tricuspid valve. The morphological diversity of two posterior leaflets is a potential risk factor for a more complicated tricuspid repair.

**Supplementary Information:**

The online version contains supplementary material available at 10.1186/s13019-022-01762-5.

## Introduction

Several studies have focused on the anatomy and pathophysiology of secondary tricuspid regurgitation (TR) associated with left heart disease or atrial fibrillation [1, 2]. Patients with significant TR, especially that in the severe range, have a poor prognosis [3, 4]. Surgical treatment can produce excellent results in patients with severe TR that medical treatment is unable to control [4, 5]. However, patients requiring reoperation because of significant residual or recurrent TR have a poor prognosis with high mortality [6]. Therefore, it is very important to address TR during the operation and analyze the risk factors for residual TR after tricuspid valve (TV) repair [7–9].

Sakon et al. [10] recently demonstrated that the number of posterior leaflets was two or more in a half of the patients analyzed in their study. However, no studies have shown that morphological diversity of the TV affects the outcomes and surgical techniques of TV repair. The present study was performed to investigate the effect of morphological diversity of the TV on the difficulty of controlling TR during TV repair.

## Materials and methods

This study was approved by the Osaka City University Ethical Review Board (approval no. 3556). Informed consent was obtained from all patients using an opt-out method.

From April 2016 to November 2020, 187 patients were diagnosed with secondary TR associated with left heart disease and underwent TV repair. Indication of TV repair was followed the 2020 Japan JCS/JSCS/JATS/JSVS Guideline [11].

**Fig. 1:**
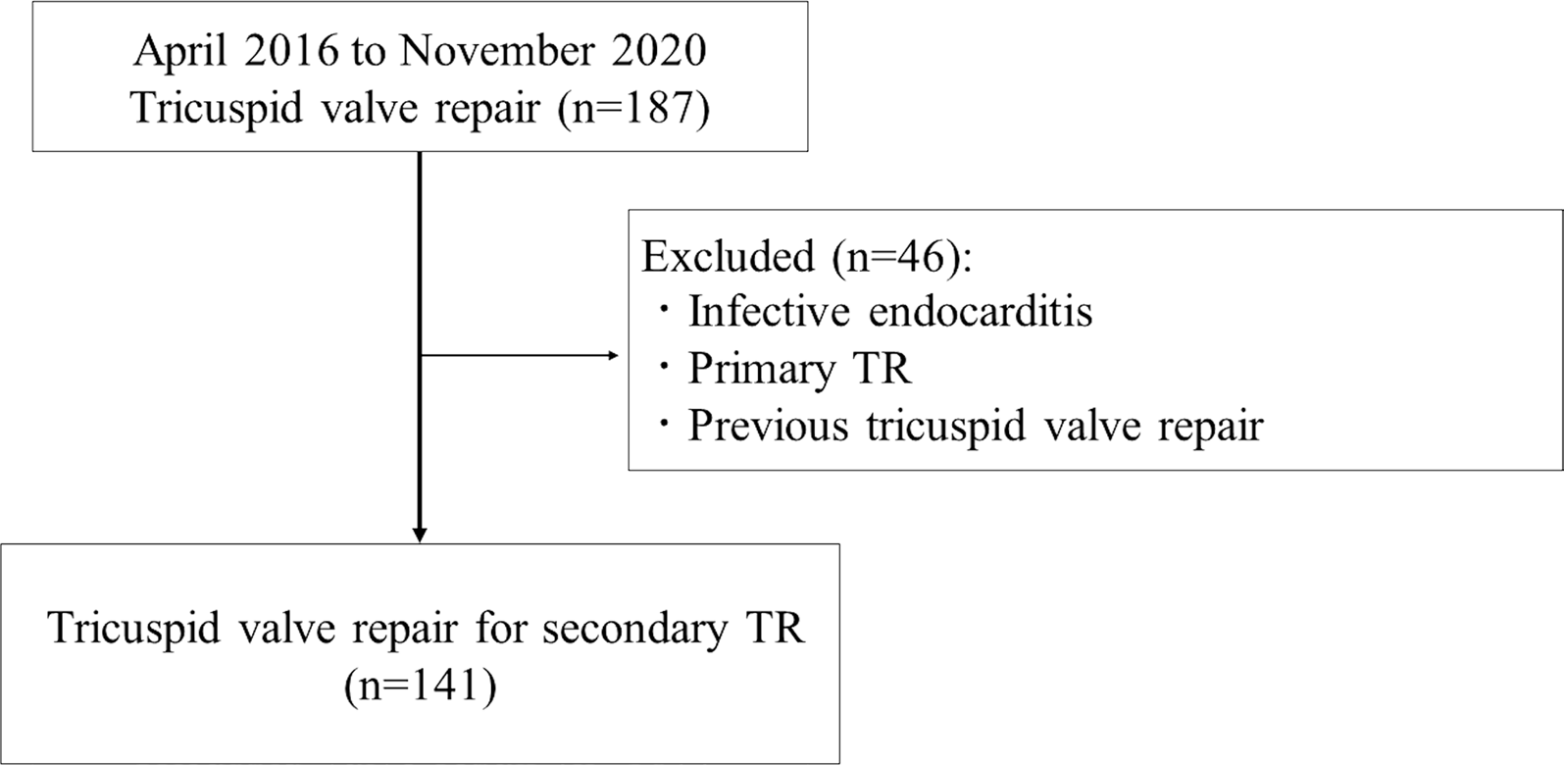
Flow diagram of patient selection. *TR*, tricuspid regurgitation

All patients underwent tricuspid annuloplasty using a Carpentier-Edwards Physio Tricuspid Annuloplasty Ring (Edwards Lifesciences, Irvine, CA, USA) or Tailor Flexible Annuloplasty Ring (Abbott, Menlo Park, CA, USA). The exclusion criteria were infective endocarditis and primary TR. Patients without perioperative echocardiographic data and patients who underwent re-do TV repair were also excluded. Finally, we analyzed 141 patients with secondary TR due to TV annular dilatation (Fig. 1).

We divided the patients into two groups: Group 1 comprised patients who underwent only ring annuloplasty for TR, and Group 2 comprised patients who required additional approximation of leaflet edges (edge-to-edge repair) with ring annuloplasty to reduce residual TR. We retrospectively examined the patients’ background data, preoperative transthoracic echocardiography data, surgical procedures, and anatomical features of the posterior leaflets and compared these data between the two groups. We also analyzed the risk predictors of the need for additional procedures to reduce TR. Finally, we assessed preoperative and postoperative echocardiographic parameters between patients with one posterior valve leaflet and patients with two posterior valve leaflets.

## Echocardiography

All patients underwent transthoracic echocardiography using an iE33 or EPIQ system (Philips Medical Systems, Andover, MA, USA) at our echocardiography laboratory preoperatively, 1 week and 1 year postoperatively.

The TR grade was defined using a multiparametric approach, including an assessment of the color Doppler-derived jet area, the continuous wave Doppler-derived jet density and contour, and the hepatic vein flow velocity pattern [12]. TR was graded as none, trivial, mild, moderate, or severe. For the statistical analysis, these TR grades were scored as follows: none = 0, none to mild = 0.5, mild = 1, mild to moderate = 1.5, moderate = 2, moderate to severe = 2.5, and severe = 3 [13].

Continuous wave Doppler was used to obtain the TR peak velocity (m/s) and the transtricuspid systolic pressure gradient (TRPG, mmHg), which was calculated as 4V^2^ (where V is velocity). The right ventricular systolic pressure was then estimated as the sum of the estimated TRPG and right atrial (RA) pressure. The RA pressure was estimated as follows: an inferior vena cava diameter of ≤ 2.1 cm that collapsed by ≥ 50% when the patient sniffed was considered to indicate a normal RA pressure of 3 mmHg, whereas an inferior vena cava (IVC) diameter of > 2.1 cm that collapsed by < 50% when the patient sniffed was considered to indicate a high RA pressure of 15 mmHg. When the IVC diameter and collapse did not fit this paradigm, an intermediate value of 8 mmHg was assigned [14]. An estimated right ventricular systolic pressure of > 40 mmHg was considered indicative of pulmonary hypertension [15]. Tricuspid annular diameter was measured at end-diastole and annular diameter of > 40 mm or 21 mm/m^2^ was considered significant annulus dilatation [11]. The RA dimension, left ventricular (LV) end-diastolic dimension, LV end-systolic dimension, LV ejection fraction, and left atrial (LA) dimension were measured according to the established guidelines [14, 16].

### Surgical technique

**Fig. 2:**
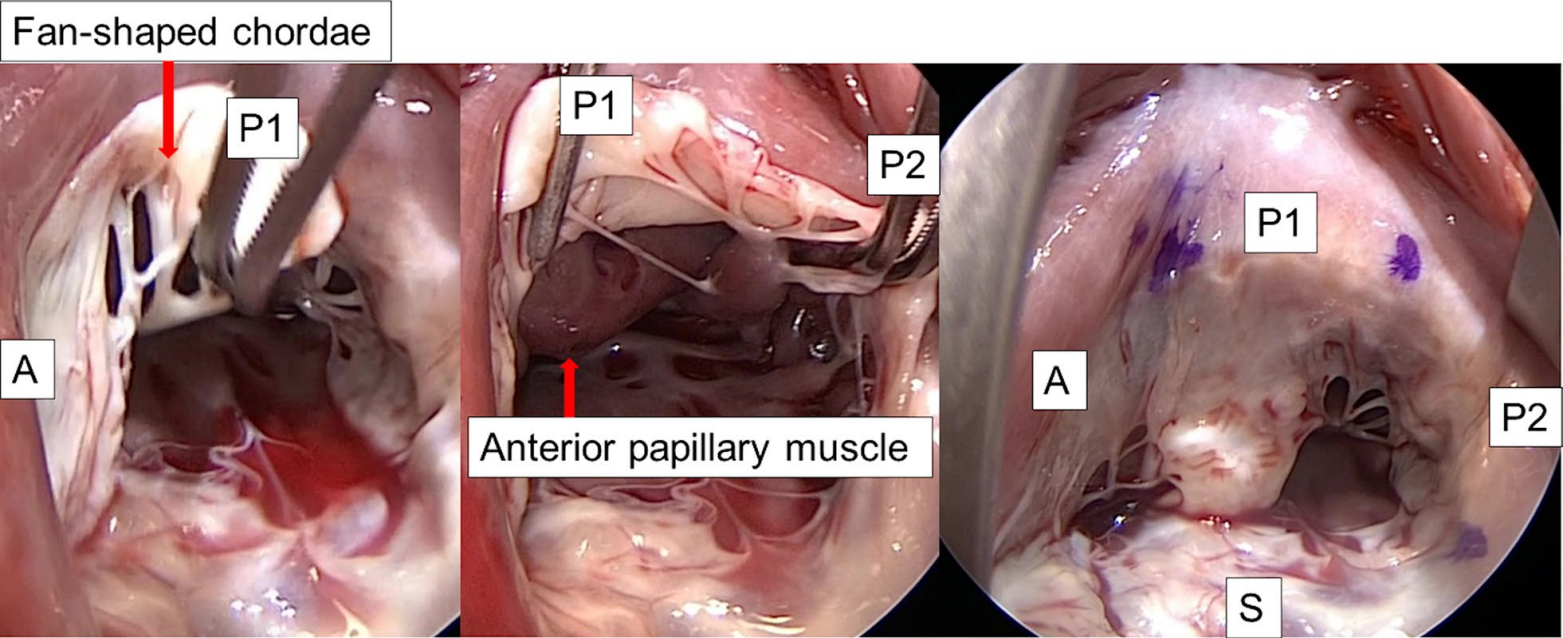
Operative findings of tricuspid valve with two posterior leaflets. *A*, anterior leaflet; *P1* and *P2*, posterior leaflets; *S*, septal leaflet

In all patients, cardiopulmonary bypass was established by ascending aortic cannulation and bicaval venous drainage through a median sternotomy. TV repair was performed concomitantly with aortic valve replacement or a mitral valve procedure in all cases. After the aortic or mitral valve procedure, we performed TV repair under cardiac arrest. The number of leaflets was determined according to the definition established by Silver et al. [17]: The commissure is defined as an indentation of the leaflets by fan-shaped chordae, the fan-shaped chordae forming the anteroposterior commissure arise from the anterior papillary muscle, and the posteroseptal commissure is defined by the fan-shaped chordae, which arise from the most medially placed papillary muscle on the posterior wall [10, 17] (Fig. 2).

**Fig. 3:**
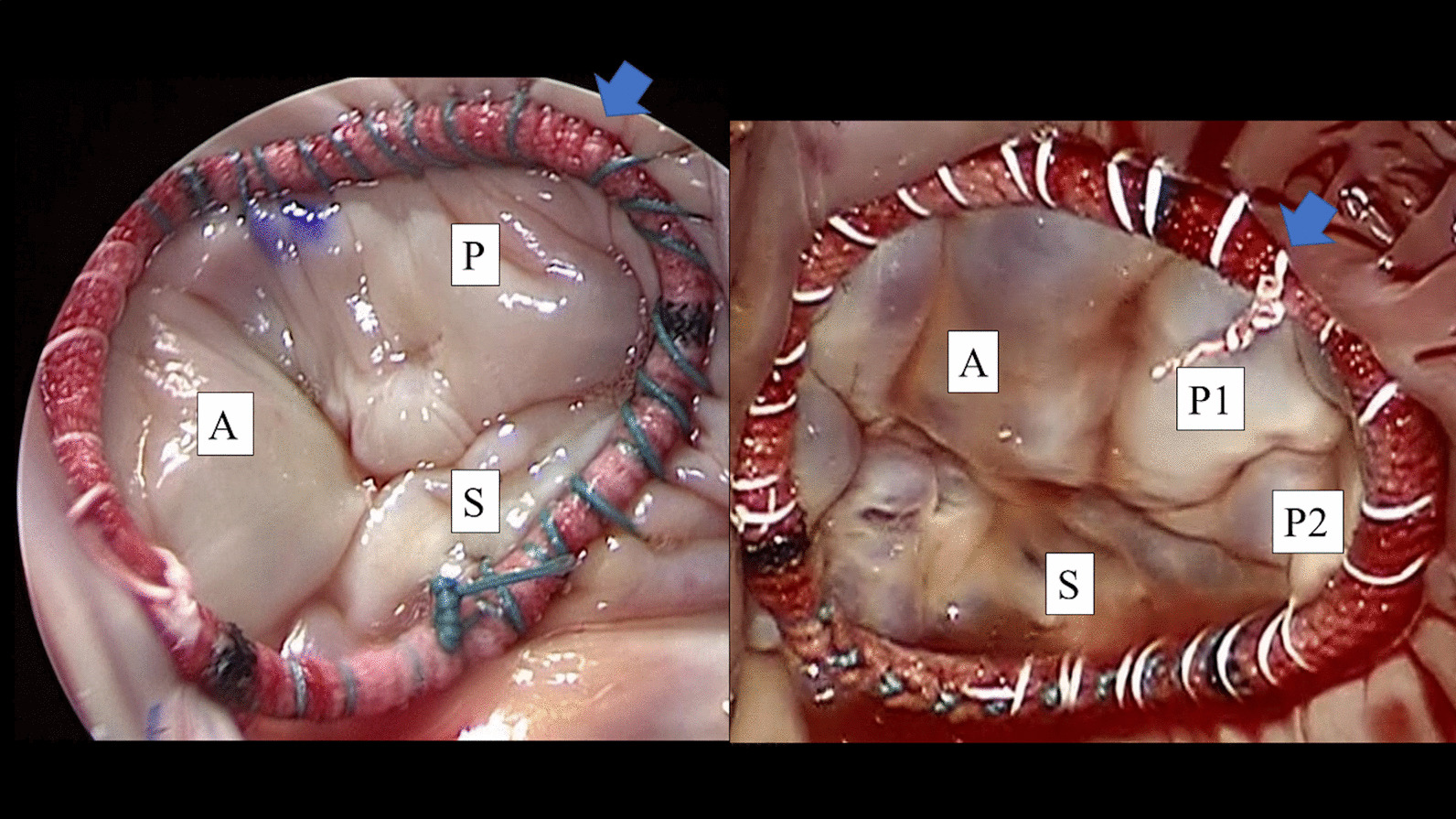
The Tailor ring was sutured with the running suture technique. We avoided placing sutures around the septal leaflet’s annulus near the atrioventricular node. We confirmed significant coaptation of each valve by the saline injection test. The blue arrow indicates the shoulder point. *A*, anterior leaflet; *P*, posterior leaflet; *S*, septal leaflet

All patients underwent tricuspid ring annuloplasty using either 2–0 polyester interrupted sutures for the Carpentier-Edwards Physio Tricuspid Annuloplasty Ring or 2–0 polyester running sutures for the Tailor Flexible Annuloplasty Ring. To prevent atrioventricular node injury, we avoided placing sutures around the septal leaflet’s annulus near the atrioventricular node when using the Tailor Flexible Annuloplasty Ring (Fig. 3). The Carpentier-Edwards Physio Tricuspid Annuloplasty Ring or the Tailor Flexible Annuloplasty Ring was used according to the surgeon’s preference. The ring size was determined comprehensively by measuring the area of the anterior leaflet or annular distance of the septal leaflet using the sizers for each ring.

**Fig. 4:**
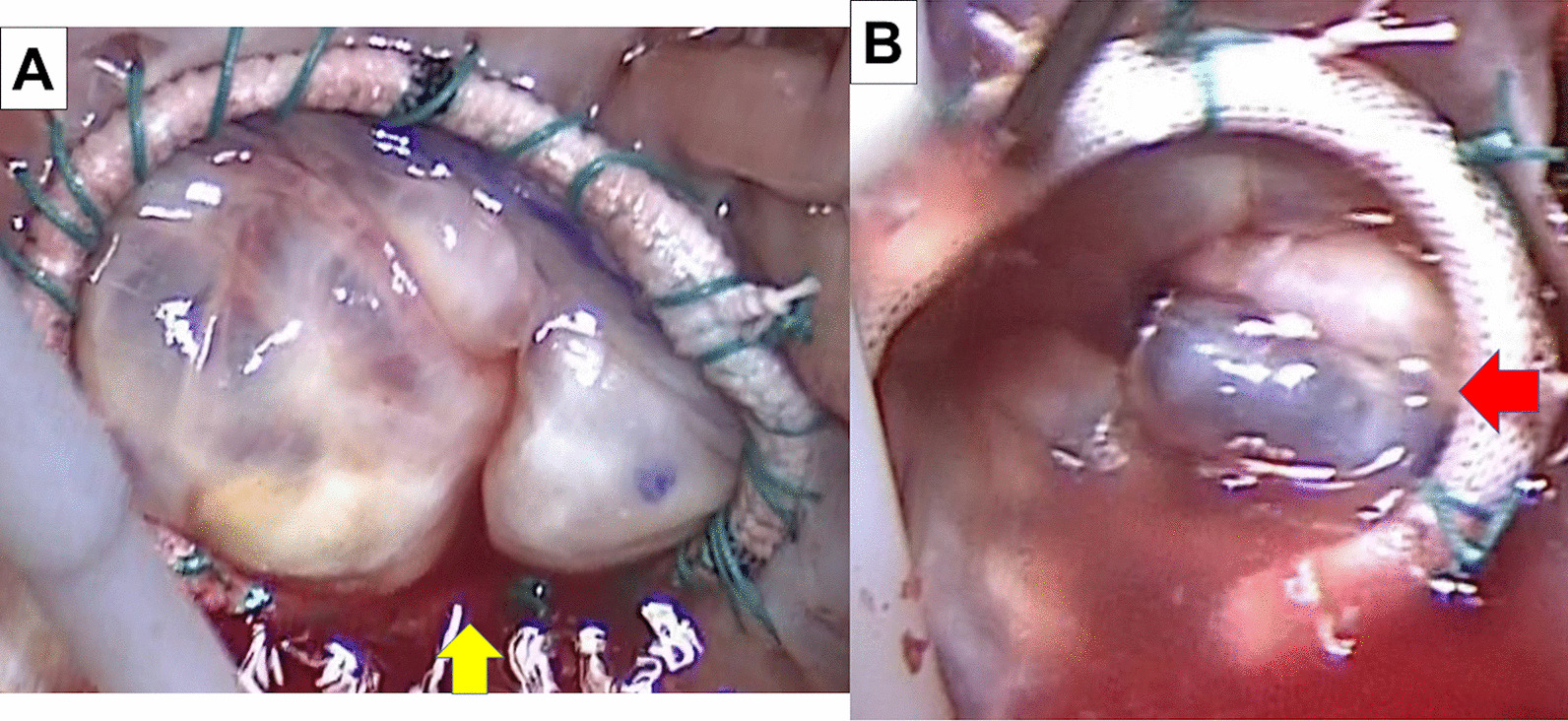
saline test after tricuspid valve repair. **a** Motion of septal leaflet was restricted and all leaflets could not have same coaptation height. **b** Only one posterior leaflet raised and other leaflets did not get adequate tension. There were gaps between a posterior leaflet and other leaflets. The yellow arrow indicated restricted septal leaflet. The red arrow indicated different height of posterior leaflet

Upon completion of the ring annuloplasty, we checked for residual TR using the saline test. During the saline test, a surgeon compressed the pulmonary artery by hand from the outside to fill the right ventricle sufficiently. Results of the saline test was classed as follows; (a) good shape (The height of all TV leaflets was aligned. All leaflets had adequate tension and coaptation.) with no leakage (Fig. 3), (b) good shape with leakage, and (c) poor shape (The height of some TV leaflets was different and tension of leaflets was insufficient.) with leakage (Fig. 4). We judged (c) as residual TR. If residual TR between the leaflets was found, we performed additional techniques to approximate leaflet edges (edge-to-edge repair) (Additional file 1: Video).

After weaning from cardiopulmonary bypass, TR was checked by intraoperative transesophageal echocardiography.

All operations and intraoperative evaluations were performed by the same surgical team.

### Statistical analysis

Descriptive statistics for categorical variables are reported as absolute value and percentage, and continuous variables are shown as mean and standard deviation. Categorical data were compared using the chi-square test. Continuous variables were compared using the Wilcoxon signed rank test.

We analyzed independent determinants of the additional procedure by performing univariate logistic regression analysis and subsequent multivariate logistic regression analysis, with the p values for entry into and removal from the model set at 0.05 and 0.10, respectively. All analyses were conducted with JMP version 13.0 (SAS Institute Inc., Cary, NC, USA), and a *p* value of < 0.05 was considered statistically significant.

## Results

### Patient’s profiles (Table 1)

**Table 1:** Patient characteristics

	Group 1 (n = 109)	Group 2 (n = 32)	*p* value
Preoperative characteristics			
Male sex	63 (58)	14 (44)	0.16
Age, years	71 ± 9.2	72 ± 10	0.51
Body surface area, m^2^	1.6 ± 0.2	1.5 ± 0.18	0.22
NYHA class ≥ II heart failure	96 (88)	30 (94)	0.33
Comorbidities			
Atrial fibrillation	73 (67)	24 (75)	0.38
Systemic hypertension	60 (55)	21 (66)	0.28
Dyslipidemia	23 (21)	11 (34)	0.13
Diabetes mellitus	16 (15)	8 (25)	0.19
Cerebrovascular event	21 (19)	9 (28)	0.29
Ischemic heart disease	18 (17)	10 (31)	0.077
Chronic renal failure	35 (32)	14 (44)	0.23
COPD	7 (6.4)	2 (6.3)	0.97
Preoperative transthoracic echocardiographic data			
RA minimum (4-chamber view), mm	42 ± 11	45 ± 11	0.11
RA maximum (4-chamber view), mm	61 ± 13	65 ± 17	0.32
LVEF, %	55 ± 11	55 ± 14	0.36
LVDd (parasternal long-axis view), mm	51 ± 8.3	50 ± 9.2	0.44
LVDs (parasternal long-axis view), mm	34 ± 8.9	33 ± 9.5	0.6
LA (parasternal long-axis view), mm	52 ± 10	55 ± 13	0.58
Pre TR score	1.6 ± 0.7	2.1 ± 0.78	0.0046
TRPG, mmHg	32 ± 13	37 ± 16	0.13
PH (estimated RVSP of > 40 mmHg)	41 (38)	19 (61)	0.019
TV diameter, mm/m^2^	24 ± 4.7	24 ± 4.3	0.69

Group 1: Patients who underwent ring annuloplasty alone

Group 2: Patients who required additional edge-to-edge repair with ring annuloplasty

Data are presented as n (%) or mean ± standard deviation

*NYHA* New York Heart Association, *COPD* chronic obstructive pulmonary disease, *RA* right atrium, *LVEF* left ventricular ejection fraction, *LVDd* left ventricular diastolic dimension, *LVDs* left ventricular systolic dimension, *LA* left atrium, *Pre TR score* preoperative tricuspid regurgitation score, *TRPG* transtricuspid pressure gradient, *PH* pulmonary hypertension (estimated right ventricular systolic (*RSVP*) pressure > 40 mmHg), *TV* tricuspid valve

A total of 32 (23%) patients needed an additional procedure with ring annuloplasty to reduce TR. There was no significant difference in the patients’ preoperative background data between the two groups.

There was no statistically significant difference in the cardiac dimension, the TRPG and the TV diameter between the two groups. The preoperative TR score was higher (2.1 ± 0.78 vs. 1.6 ± 0.7; *p* = 0.0046), and more patients had pulmonary hypertension [19 (61%) vs. 41 (38%); *p* = 0.019] in Group 2 than in Group 1, respectively.

### Surgical data (Table 2)

**Table 2:** Surgical data

	Group 1 (n = 109)	Group 2 (n = 32)	*p* value
Morphological diversity			
Valve with two posterior leaflets	36 (33)	20 (63)	0.003
Annuloplasty ring			
Carpentier-Edwards physio tricuspid Annuloplasty ring	76 (70)	26 (81)	0.19
Tailor flexible annuloplasty ring	33 (30)	6 (19)	
Ring size, mm	29 ± 1.8	29 ± 1.7	0.19
Concomitant procedures			
AVR	41 (38)	5 (16)	0.014
MVP	47 (43)	15 (47)	0.71
MVR	45 (41)	16 (50)	0.38
AVR or MVP or MVR + maze procedure	32 (29)	6 (19)	0.22

Group 1: Patients who underwent ring annuloplasty alone

Group 2: Patients who required additional edge-to-edge repair with ring annuloplasty

Data are presented as n (%) or mean ± standard deviation

*AVR* aortic valve replacement, *MVP* mitral valve repair, *MVR* mitral valve replacement

A total of 56 (40%) patients had two posterior leaflets of the TV. The proportion of patients with two posterior leaflets was higher in Group 2 than in Group 1 [20 (63%) vs. 36 (33%), respectively; *p*=0.003]. All patients underwent TV ring annuloplasty. There was no statistically significant difference in the ring type or ring size between the two groups. Aortic valve replacement was more frequently performed as a concomitant procedure in Group 1 than in Group 2 [41 (38%) vs. 5 (16%), respectively; *p*=0.014].

### Additional procedures

Edge-to-edge repair was performed in all patients in Group 2.

There were 25 cases with one edge-to-edge repair in Group 2 (4 cases with edge-to-edge at anterior and posterior leaflets, 11 cases at posterior and septal leaflets, 9 cases at anterior and septal leaflets, and 1 case at posterior and posterior leaflets). There were 6 cases with 2 edge-to-edge repairs in Group 2 (3 cases with edge to edge at the anterior–posterior leaflet and posterior-septal leaflet, 2 cases at the anterior-septal leaflet and posterior-septal leaflet, and 1 case at the anterior-septal leaflet and posterior-posterior leaflet). One patient in Group 2 underwent three edge-to-edge repairs at the anterior-septal leaflet, anterior–posterior leaflet, and posterior-septal leaflet.

In all patients in both groups, TR was controlled, confirmed by the intraoperative saline test. The 1-week postoperative TR score in all 141 patients decreased, confirmed by echocardiography. TR score improved from 1.6 ± 0.7 to 0.7 ± 0.32 (*p* < 0.0001) in Group 1 and from 2.1 ± 0.78 to 0.88 ± 0.49 (*p* < 0.0001) in Group 2.

### Postoperative transthoracic echocardiographic data (Table 3)

**Table 3:** Postoperative transthoracic echocardiographic data

	Group 1 (n = 109)	Group 2 (n = 32)	*p* value
RA minimum (4-chamber view), mm	36 ± 6.1	36 ± 6.7	0.74
RA maximum (4-chamber view), mm	49 ± 8.5	47 ± 8.4	0.54
LVEF, %	52 ± 11	52 ± 12	0.81
LVDd (parasternal long-axis view), mm	48 ± 7.4	47 ± 7.7	0.32
LVDs (parasternal long-axis view), mm	32 ± 8.2	32 ± 7.9	0.72
LA (parasternal long-axis view), mm	46 ± 8.5	46 ± 8.4	0.46
Post TR score	0.7 ± 0.32	0.88 ± 0.49	0.043
TRPG, mmHg	19 ± 11	24 ± 14	0.05
PH (estimated RVSP > 40 mmHg)	0 (0)	0 (0)	

Group 1: Patients who underwent ring annuloplasty alone

Group 2: Patients who required additional edge-to-edge repair with ring annuloplasty

Data are presented as n (%) or mean ± standard deviation

*RA* right atrium, *LVEF* left ventricular ejection fraction, *LVDd* left ventricular diastolic dimension, *LVDs* left ventricular systolic dimension *LA* left atrium, *Pre TR score* preoperative tricuspid regurgitation score, *TRPG* transtricuspid pressure gradient, *PH* pulmonary hypertension (estimated right ventricular systolic pressure (*RSVP*) > 40 mmHg)

All patients underwent transthoracic echocardiography 1 week after surgery. There was no statistically significant difference in the cardiac dimensions between the two groups. The postoperative TR score (0.88 ± 0.49 vs. 0.7 ± 0.32; *p* = 0.043) and the TRPG (24 ± 14 mmHg vs. 19 ± 11 mmHg; *p* = 0.05) were higher in Group 2 than in Group 1, respectively.

### Predictors of additional procedures (Table 4)

**Table 4:** Univariate and multivariate logistic regression analyses of the risk predictors of requiring additional procedures

	Univariate analysis			Multivariate analysis		
	OR	95% CI	*p* value	OR	95% CI	*p* value
Model using echocardiographic data						
Pre TR score	2.3	1.3–4.1	0.0025	1.8	1.0–3.4	0.042
PH (estimated RVSP of > 40 mmHg)	2.6	1.2–6.0	0.021	1.9	0.79–4.6	0.15
RA minimum (4-chamber view)	1.0	0.99–1.1	0.14			
RA maximum (4-chamber view)	1.0	0.99–1.0	0.28			
TRPG	1.0	0.99–1.0	0.20			
LVDd (parasternal long-axis view)	0.99	0.94–1.0	0.57			
LVDs (parasternal long-axis view)	0.98	0.95–1.0	0.61			
TV diameter, mm/m^2^	0.97	0.89–1.1	0.44			
Model using clinical data						
Valve with two posterior leaflets	3.4	1.5–7.7	0.0036	2.6	1.1–6.1	0.033
Male sex	0.56	0.26–1.3	0.16			
Body surface area	0.22	0.026–1.9	0.16			
Age	1.0	0.96–1.1	0.75			
NYHA class ≥ II heart failure	2.0	0.43–9.5	0.33			
Atrial fibrillation	1.5	0.61–3.6	0.38			
Dyslipidemia	2.0	0.82–4.6	0.13			
Ischemic heart disease	2.3	0.93–5.7	0.078			

*OR* odds ratio, *CI* confidence interval, *Pre TR score* preoperative tricuspid regurgitation score, *PH* pulmonary hypertension (estimated right ventricular systolic pressure > 40 mmHg), *RVSP* right ventricular systolic pressure, *RA* right atrium, *TRPG* transtricuspid pressure gradient, *LVDd* left ventricular diastolic dimension, *LVDs* left ventricular systolic dimension, *TV* tricuspid valve, *NYHA* New York Heart Association

Table 4 shows the results of the univariate and multivariate logistic regression analyses to identify the predictors of the need for additional procedures. In the model using echocardiographic data and clinical data, the preoperative TR score (odds ratio, 1.8; 95% confidence interval, 1.0–3.4; *p* = 0.042) and the presence of a TV with two posterior leaflets (odds ratio, 2.6; 95% confidence interval, 1.1–6.1; *p* = 0.033) were independent predictors of additional procedures, according to the multivariate analysis.

### Comparison of echocardiographic parameters for each number of posterior leaflets (Table 5)

**Table 5:** Perioperative echocardiographic parameters for each number of valve leaflets

	One posterior valve leaflet	Two posterior valve leaflets	*p* value
	n = 85	n = 56	
Preoperative data			
RA minimum (4-chamber view), mm	42 ± 11	43 ± 11	0.51
RA maximum (4-chamber view), mm	62 ± 13	63 ± 16	0.86
LVEF, %	53 ± 12	58 ± 9.2	0.014
LVDd (parasternal long-axis view), mm	51 ± 8.2	52 ± 8.9	0.34
LVDs (parasternal long-axis view), mm	33 ± 9.1	34 ± 8.9	0.76
LA (parasternal long-axis view), mm	52 ± 10	54 ± 12	0.25
Pre TR score	1.6 ± 0.67	1.9 ± 0.81	0.032
TRPG, mmHg	34 ± 15	32 ± 12	0.61
PH (estimated RVSP > 40 mmHg)	32 (38)	28 (51)	0.12
TV diameter, mm/m^2^	24 ± 4.5	25 ± 4.7	0.42
Postoperative data			
RA minimum (4-chamber view), mm	36 ± 6.3	35 ± 6.3	0.89
RA maximum (4-chamber view), mm	48 ± 8.0	49 ± 9.2	0.46
LVEF, %	52 ± 12	53 ± 10	0.82
LVDd (parasternal long-axis view), mm	48 ± 7.3	48 ± 7.8	0.96
LVDs (parasternal long-axis view), mm	32 ± 8.5	32 ± 7.6	0.97
LA (parasternal long-axis view), mm	45 ± 7.7	46 ± 9.5	0.77
Post TR score	0.76 ± 0.36	0.71 ± 0.4	0.58
TRPG, mmHg	21 ± 11	19 ± 12	0.41

	n = 49	n = 29	
One year after operation data			
RA minimum (4-chamber view), mm	36 ± 6.0	39 ± 5.7	0.1
RA maximum (4-chamber view), mm	47 ± 8.7	50 ± 8.3	0.12
LVEF, %	54 ± 9.6	55 ± 7.7	0.76
LVDd (parasternal long-axis view), mm	46 ± 6.5	46 ± 5.7	0.89
LVDs (parasternal long-axis view), mm	30 ± 8.0	30 ± 5.2	0.34
LA (parasternal long-axis view), mm	46 ± 8.4	47 ± 12	0.95
Post TR score	0.7 ± 0.3	0.95 ± 0.43	0.0075
TRPG, mmHg	21 ± 8.8	26 ± 14	0.1

Data are presented as n (%) or mean ± standard deviation

*RA* right atrium, *LVEF* left ventricular ejection fraction, *LVDd* left ventricular diastolic dimension, *LVDs* left ventricular systolic dimension, *LA* left atrium, *Pre TR score* preoperative tricuspid regurgitation score, *Post TR score* postoperative tricuspid regurgitation score, *TRPG* transtricuspid pressure gradient, *PH* pulmonary hypertension (estimated right ventricular systolic pressure (*RSVP*) > 40 mmHg), *TV* tricuspid valve

There was no significant difference between patients with one posterior valve leaflet and patients with two posterior valve leaflets regarding cardiac dimension in perioperative periods. Patients with two posterior valve leaflets had better cardiac LV contraction than those with one posterior valve leaflet before operation (58% ± 9.2% vs. 53% ± 12%, respectively; *p* = 0.014). There were also no significant differences in the TRPG, the number of patients with pulmonary hypertension and the TV diameter. However, the presence of two posterior valve leaflets was associated with a higher preoperative TR score than one posterior valve leaflet (1.9 ± 0.81 vs. 1.6 ± 0.67, respectively; *p* = 0.032). Regarding echocardiographic parameters after operation, there was no difference of TR score just after surgery between groups, but two posterior leaflets cases had higher TR score than one posterior leaflet cases one year after operation (0.95 ± 0.43 vs. 0.7 ± 0.3, respectively; *p* = 0.0075).

## Discussion

In the present study, a TV with two posterior leaflets was an independent predictor of additional procedures. The TV consists of multiple posterior leaflets in about half of patients, and the morphological diversity of these leaflets has attracted attention among researchers [10, 17, 18].

We consider that postoperative TR can be divided into two types: TR observed immediately after surgery (residual TR) and TR that worsens during follow-up despite the fact that it was controlled immediately after surgery (recurrent TR). Fukuda et al. [9] reported that residual TR soon after the operation causes volume overloading of the right ventricle and further right ventricular dilatation and dysfunction, resulting in worsening TR. Therefore, we consider that controlling the residual TR grade during the perioperative period is crucial to avoid the development of later TR.

Upon completion of the ring annuloplasty, we routinely checked TV by the saline test. If residual TR was found, we performed additional edge-to-edge repair at the sites of the leakage. Other techniques, such as artificial chords for prolapse cases, appear common, and Salihi et al. reported that artificial neochordae implantation was effective as an adjunct procedure [19]. We selected edge-to-edge repair to simplify and unify the surgical technique.

Although we could control TR with ring annuloplasty alone in most patients, additional sutures were necessary to control residual TR in some patients. The locations and numbers of edge-to-edge repairs varied among the patients according to the features of the residual TR. Thus, we investigated the crucial causes of complicated TV repair in our study.

The surgical technique to control TR depends upon the mechanism of TR, such as annular dilatation, prolapse or tethering of the leaflets, or right ventricular dilatation. We suspected that morphological differences of the TV may make it difficult to control TR during surgery. Therefore, we focused on TV morphology and the complexity, to control TR. We set the endpoint as whether additional procedures were needed during the operation. According to the univariate and multivariate logistic regression analyses, patients with two posterior valve leaflets required additional procedures for reduction of TR. Moreover, patients with two posterior valve leaflets had higher TR score before operation and one year after operation than those with one posterior valve leaflet. There was no difference in the size of the TV regardless of the number of posterior leaflets. Considering these results, we concluded that the presence of two posterior valve leaflets itself has the potential to increase regurgitation, resulting in the need for complex procedures in TV repair.

When we classify the morphological diversity of the TV leaflets, identification of the commissure between the anterior and posterior leaflets is important. When the TV has two posterior leaflets, the commissural cleft between the anterior and posterior leaflets is sometimes misidentified as a deep cleft of the anterior leaflet. This is because most surgeons do not observe fan-shaped chordae arising from the anterior papillary muscle and do not recognize that half of patients have a TV with two posterior leaflets. When surgeons misinterpret the commissure between the anterior and posterior leaflets, they might choose a smaller annuloplasty ring and adjust the marker on the ring to an inappropriate position, especially in patients with two posterior leaflets. A smaller ring might deform TV annulus and impose more stress on suture lines. We measured both the distance between the commissure of the septal leaflet and the area of the anterior leaflet for proper sizing of annuloplasty ring, resulting in selection of larger ring. To avoid deformation of the TV, we applied the “shoulder point fitting method” of proportional annuloplasty in all patients [18]. The shoulder point is defined as the 2-o’clock position of the TV annulus, where the TV annulus is more widely dilated. This technique has a lower risk of TV annulus deformation because annuloplasty is performed toward the shoulder point [18]. This method also supports patients with multiple posterior leaflets. We are convinced that proportional annuloplasty was achieved in all patients regardless of the number of posterior leaflets in this study.

### Limitations

Our study has several limitations. First, it was retrospective and involved a small number of patients. Therefore, the differences in the patients’ backgrounds between the two groups could not be statistically adjusted. In addition, the reason why concomitant AVR was present more commonly in group 1 was unclear. These issues should be addressed in future prospective studies containing larger numbers of patients. Second, we used two types of rings and two types of suturing techniques when performing TV repair. The type of ring and technique should be unified in future studies. Third, we could not clarify the mechanism of residual TR in patients with posterior leaflets in spite of using shoulder point fitting method. Finally, because our study was limited to the perioperative results, further evaluation should also involve the mid-term follow-up period.

## Conclusion

The morphological diversity of two posterior leaflets is a potential risk factor that makes TV repair complicated and that leaves residual TR.

## Supplementary Information

**Additional file 1**.** Video**: The TV has two posterior leaflets. After ring annuloplasty, we checked TV by saline test. A posterior leaflet raised and there were gaps between the posterior leaflet and other leaflets. A 5-0 polypropylene suture was placed to approximate at posterior and posterior leaflets.

## Data Availability

The datasets used or analyzed during the current study are available from the corresponding author on reasonable request.

## Abbreviations

TR: Tricuspid regurgitation
TV: Tricuspid valve
TRPG: Transtricuspid systolic pressure gradient
RA: Right atrial
IVC: Inferior vena cava
LV: Left ventricular
LA: Left atrial
